# Supplementary material for: Self-mixing in microtubule-kinesin active fluid from nonuniform to uniform distribution of activity
Source: Nat Commun. 2022 Nov 2;13:6573. doi: 10.1038/s41467-022-34396-1 (PMC9630547; doi:10.1038/s41467-022-34396-1)
Supplement: Supplementary file 1 — Supplementary Information [file 41467_2022_34396_MOESM1_ESM.pdf]

Supplementary Information:
Self-mixing in microtubule-kinesin active fluid from nonuniform to uniform distribution of activity

Teagan E Bate[1], Megan E Varney[2], Ezra H Taylor[1], Joshua H Dickie[1], Chih-Che Chueh[3], Michael M Norton[4], and Kun-Ta Wu[1,5,6,*]

[1] Department of Physics, Worcester Polytechnic Institute, Worcester, Massachusetts 01609, USA
[2] Department of Physics, New York University, New York, New York 10003, USA
[3] Department of Aeronautics and Astronautics, National Cheng Kung University, Tainan 701, Taiwan
[4] School of Physics and Astronomy, Rochester Institute of Technology, Rochester, New York 14623, USA
[5] Department of Mechanical Engineering, Worcester Polytechnic Institute, Worcester, Massachusetts 01609, USA
[6] The Martin Fisher School of Physics, Brandeis University, Waltham, Massachusetts 02454, USA
[*]Corresponding: kwu@wpi.edu

Table of Contents

Supplementary Figure 1 2

Supplementary Note 1: Estimation of ATP molecular diffusion coefficient in crosslinked microtubule networks 3

Supplementary Figure 2 4

Supplementary Note 2: Effect of flow speed-ATP relation on active-inactive interface progression in the Fick's law-based model 5

Supplementary Figure 3 7

Supplementary Figure 4 8

Supplementary Note 3: Analytical expressions for active-inactive interface progression 9

Supplementary Note 4: Effect of sample container height on the correlation lengths and times of flow velocities 10

Supplementary Figure 5 11

Supplementary Note 5: Mixing kinematics of activity-uniform active fluid 12

Supplementary Figure 6 12

Supplementary Note 6: Network melting mechanism may slow progression of active-inactive interface 13

Supplementary Figure 7 15

Supplementary Figure 8 16

Supplementary Figure 9 17

Supplementary Figure 10 18

Supplementary Movies 19

Supplementary References 20

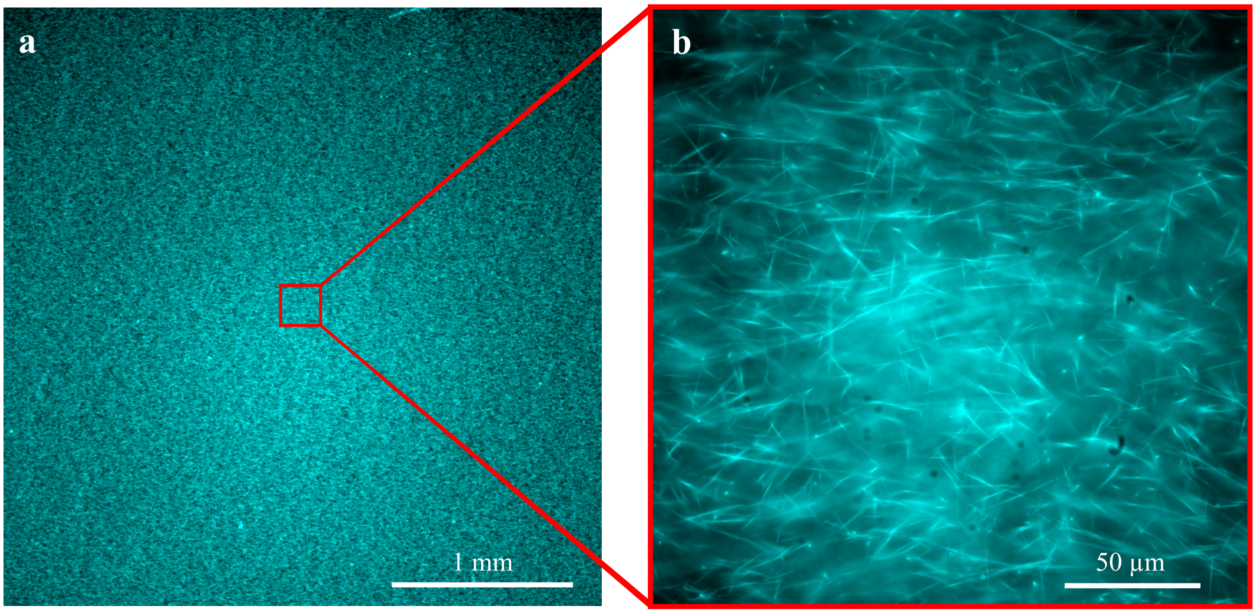

Supplementary Figure 1: (Experimental results) Microtubules in inactive fluid loaded into the flow cell before UV activation. (a) Micrograph of microtubules at millimeter scale. (b) Micrograph of microtubules at micrometer scale, showing an initial dominant alignment of microtubules parallel to the long edge of the flow cell (horizontal direction) caused by the shear flow induced by pipetting the sample into the cell.[1]

Supplementary Note 1: Estimation of ATP molecular diffusion coefficient in crosslinked microtubule networks

ATP molecular diffusion plays an important role in our characterization of active-inactive fluid mixing. For example, we characterize the dominant mechanism in active transport (i.e., diffusion or convection) by introducing the Péclet number, Pe (Figs. 4 & 5),[2,3] which depends on the molecular diffusion coefficient of ATP. We also adopt Fick's law (Eq. 1) to model the dispersion of ATP in low Pe regimes (Fig. 3), which also requires the ATP diffusion coefficient. As such, it is important to determine the molecular diffusion coefficient of ATP. It is challenging to directly observe the diffusion of ATP, but fluorescein can be directly visualized, and thus we analyze the molecular diffusion coefficient of fluorescein in the inactive microtubule network as the basis for estimating the molecular diffusion coefficient of ATP in our active fluid system.

We observe and characterize the dispersion of fluorescein near the interface of caged and UV-uncaged fluorescein suspended in inactive microtubule-kinesin fluid (Fig. 5a, Supplementary Figure 2a) and compare the observation with the solution of Fick's law (Eq. 1) to extract the corresponding molecular diffusion coefficient. For each micrograph, we average the gray values vertically to determine the gray value profile (Supplementary Figure 2b). We assume that the fluorescein concentration is proportional to the gray values, and thus the profiles of gray values should satisfy the Fick's law equation (Eq. 1), whose solution in a boundless 1D system takes the form of a complementary error function:

$$C(x,t) = \frac{C_0}{2}\,\mathrm{erfc}\left(\frac{x - x_0}{2\sqrt{D(t - t_0)}}\right), \qquad 1$$

where C_0 is the initial concentration of the suspended molecules, erfc is the complementary error function, and D is the molecular diffusion coefficient of the suspended molecules. Thus, the profiles of gray values (G.V.) should take the form of

$$\text{G.V.}(x,t) = g_0\,\mathrm{erfc}\left(\frac{x - x_0}{2\sqrt{\Gamma(t)}}\right) + g_0', \qquad 2$$

where g_0 is the prefactor and $\Gamma \equiv D(t - t_0)$. We add the constant g_0' to represent the gray value contributed from the background light in the microscope room. Then we fit this form to the profiles of gray values with g_0, g_0', x_0, and Γ as fitting parameters (Supplementary Figure 2b inset). The fit Γ is expected to be linear to time t, so we plot Γ as a function of time t and fit Γ vs. t to $\Gamma = D(t - t_0)$ with D and t_0 as fitting parameters (Supplementary Figure 2c), which reveals that the molecular diffusion coefficient of fluorescein in our inactive microtubule-kinesin system is $D = 97.4 \pm 0.2$ μm^2/s. This analyzed diffusion coefficient is one-fifth the value reported in aqueous solution (400-600 μm^2/s).[4-7] We attribute this discrepancy to the rheologically complex environment of the inactive fluid that results from crosslinked microtubules.[8] We assume that the molecular diffusion of ATP molecules is similarly diminished in our experiments. The reported diffusion coefficient of ATP in water is 710 μm^2/s,[9] and we therefore estimate the diffusivity of ATP to be 140 μm^2/s in our models and calculations (Figs. 3-5).

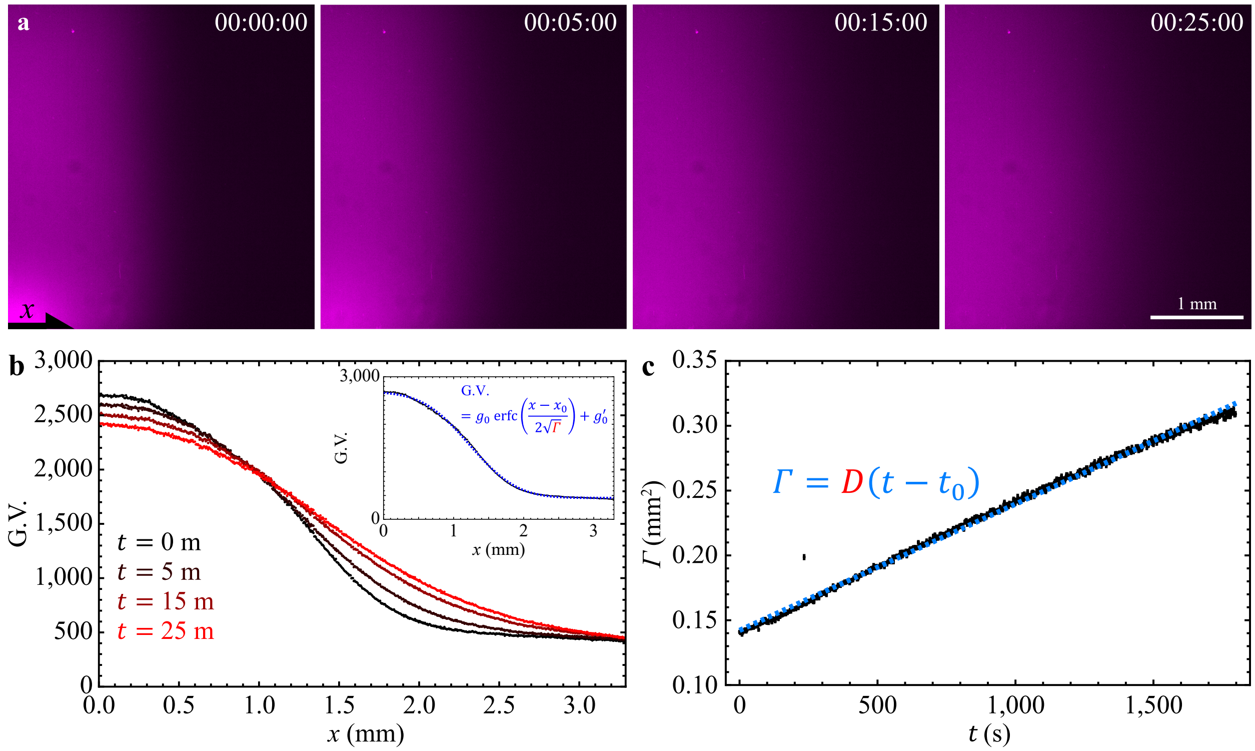

Supplementary Figure 2: (Experimental results) Analysis of molecular diffusion coefficient of fluorescein suspended in inactive microtubule-kinesin fluid. The fluorescein is caged such that it only fluoresces after exposure to ultraviolet light (Fig. 5a). (a) Micrographs of fluorescein near the edge of the ultraviolet light-exposed area. The time stamps indicate hour:minute:second. (b) Profiles of gray values in the micrographs shown in Panel a. Inset: The gray value profile of the micrograph at time 00:00:00 (black dots) is fit to a complementary error function (Supplementary Equation 2; blue dashed curve) to extract the parameter Γ which is expected to linearly increase with time t. (c) The fit parameter Γ as a function of time t (black dots) is fit to a line function (blue dashed line). The fit slope, $D = 97.4 \pm 0.2$ μm^2/s, represents the molecular diffusion coefficient of fluorescein suspended in crosslinked microtubule networks.

Supplementary Note 2: Effect of flow speed-ATP relation on active-inactive interface progression in the Fick's law-based model

We develop the Fick's law-based model (Fig. 3) to describe the experimentally observed mixing of active and inactive fluids (Fig. 2). Our model uses Michaelis-Menten kinetics to convert distribution of ATP to flow speed (Eq. 4) because flow speed is driven by microtubule motion, which in turn depends on the stepping rate of kinesin motors which follows Michaelis-Menten kinetics.[10-12] Our previous work shows that Michaelis-Menten kinetics reasonably connect flow speed of active fluid with ATP concentrations when the ATP concentration is above 100 μM.[13] Below this concentration, inactive kinesin motor dimers start to act as a crosslinkers in the microtubule network, causing the network to behave more like an elastic gel, and Michaelis-Menten kinetics fail to describe the flow speed because Michaelis-Menten kinetics is an enzyme-based model that does not consider network rheology.

As such, our adoption of Michaelis-Menten kinetics to convert ATP concentration to flow speed is an approximation for active fluid with high ATP concentrations (≳100 μM), and it is unclear how the results of our Fick's law-based model will change if we choose a different relation between ATP concentration and flow speed. Here we consider that the flow speed is connected to ATP concentration via a positive power-law exponent, $n > 0$, of the Michaelis-Menten relation:

$$\bar{v} = \bar{v}_{\mathrm{m}} \left(\frac{C}{C + K}\right)^n, \qquad 3$$

where $\bar{v}_{\mathrm{m}} = 6.2$ μm/s is the saturated mean speed of active fluid and $K = 270$ μM is the ATP concentration that leads to half of the saturated mean speed, $\bar{v}_{\mathrm{m}}/2$ (Supplementary Figure 3a).[13] Then we explore how n will affect the predicted active-inactive interface progression in terms of progression exponent γ and coefficient P_{I}.

To find γ vs. n and P_{I} vs. n, we first consider the case of initial ATP concentration $C_0 = 8$ mM and solve the 1D Fick's law equation with the same boundary and initial conditions (Eqs. 1-3) for $C(x,t)$. Then we convert ATP concentration $C(x,t)$ to flow speed $\bar{v}(x,t)$ using our power-lawed Michaelis-Menten equation (Supplementary Equation 3), which shows that increasing power law exponents n leads to a slower progression of the active-inactive interface (Supplementary Figure 3b). However, such an n-induced variation in interface progression appears not to interfere with the relation of interface displacement with time; we find that the squared interface displacement increases linearly with time across our explored power-law exponents ($n = 0.5$-10; Supplementary Figure 3c), which suggests that interface progression exponent γ will equal 1 regardless of the value of the power-law exponent n in our explored range of exponents (Supplementary Figure 3d inset). Contrarily, we find that the interface progression coefficient decreases with increasing power-law exponents (Supplementary Figure 3d). Overall, our exploration reveals that the active-inactive interface progression exponent being 1 is a consequence of the diffusion-like process of ATP dispersion. This result is insensitive to the choice of flow speed-ATP model (Supplementary Equation 3), but the interface progression coefficient varies rapidly with the model choice. Increasing the power-law exponent from 0.5 to 10 decreases the interface progression coefficient from 740 to 34 μm^2/s. Given that our experimentally measured interface progression coefficient $P_{\mathrm{I}} = 451 \pm 8$ μm^2/s for 8 mM caged ATP concentration (Fig. 3d), this also suggests that selecting $n = 1$ (or slightly larger than 1) will best match our model with the experimental results (Supplementary Figure 3d) and that Michaelis-Menten kinetics (Eq. 4) is a good approximation to connect ATP concentrations with the local flow speed of active fluid.

The calculations in our Fick's law-based model reveals that the interface progression coefficient for a given C_0 depends on the selected flow speed-ATP relation (Supplementary Figure 3a), which implies that the

ATP dependence of the interface progression coefficient $P_{\mathrm{I}}(C_0)$ should change with the selected flow speed-ATP relation as well. To investigate, we repeat the above calculation and determine the interface progression coefficients, P_{I}, as a function of initial ATP concentration, C_0, for power-law exponents n ranging from 0.5 to 10 (dots in Supplementary Figure 4a). Our analysis reveals that increasing the power-law exponents decreases P_{I} as a function of C_0 (Supplementary Figure 4a) in a similar way that it does for flow speed (Supplementary Figure 3a). Inspired by this observation, we fit each P_{I} vs. C_0 to their corresponding power-lawed Michaelis-Menten equation with the same power-law exponent n:

$$P_{\mathrm{I}} = P_{\mathrm{m}} \left(\frac{C_0}{C_0 + K_{\mathrm{P}}} \right)^n, \qquad 4$$

with P_{m} and K_{P} as fitting parameters (curves in Supplementary Figure 4a). The resulting data fit well to the equations, with overall goodness of fit $R^2 \geq 0.99$ (Supplementary Figure 4b). This suggests that the ATP dependence in the flow speed can pass to the resulting interface progression coefficient P_{I}. This analysis also shows that the consistency between the model $P_{\mathrm{I}}(C_0)$ and experimentally measured P_{I} vs. C_0 (Fig. 3d) is under the condition of $n \approx 1$, which reenforces our assertion that adopting the Michaelis-Menten equation to convert ATP concentration to active fluid flow speed is an appropriate approach for building a coarse-grained model that matches the experimental results.

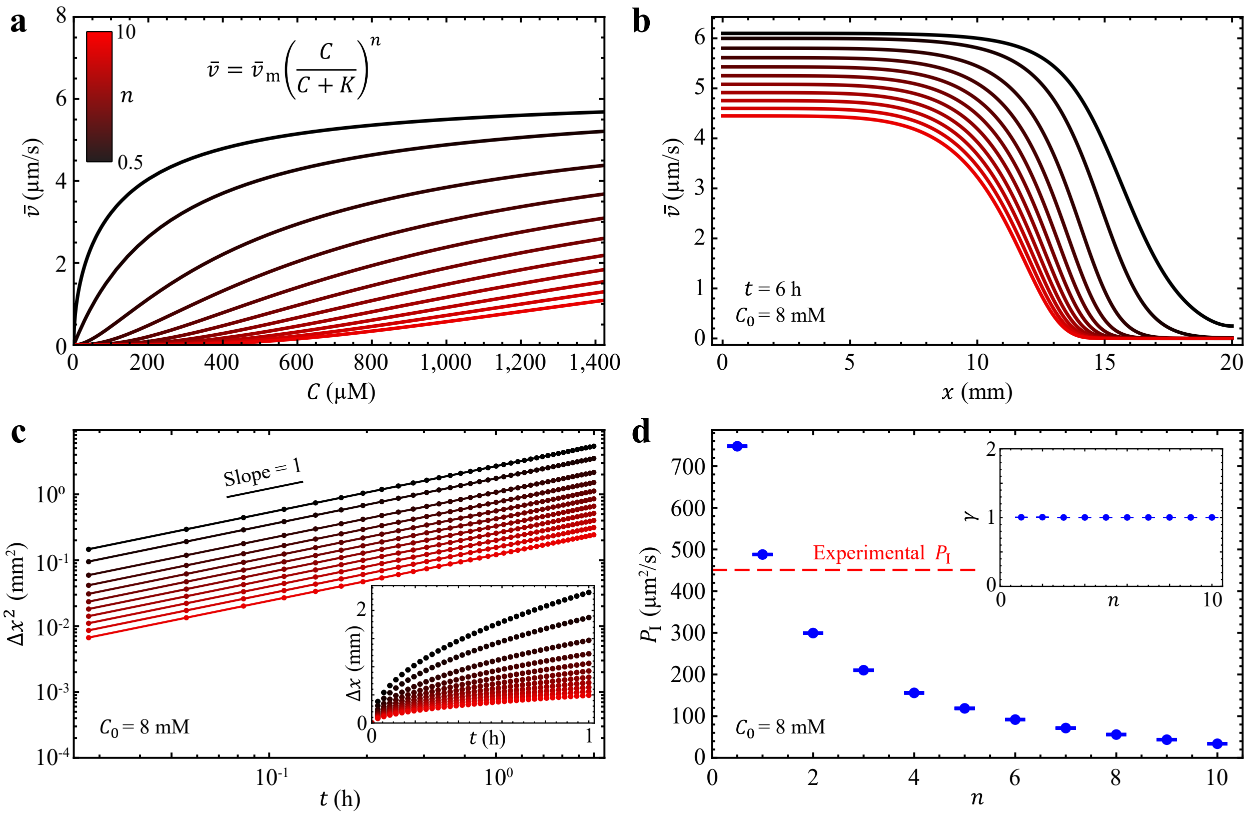

Supplementary Figure 3: (Modeling results) Exploration of the Fick's law-based model with various flow speed-ATP relations. The Fick's law-based model shows that the power-law exponent, n, in flow speed-ATP relation $\bar{v} = \bar{v}_m\{[ATP]/([ATP]+K)\}^n$, influences the interface progression coefficients but not the interface progression exponents. (a) Plot of flow speed of active fluid as a function of ATP concentration, $\bar{v} = \bar{v}_m\{[ATP]/([ATP]+K)\}^n$, with different power-law exponents n, where $\bar{v}_m = 6.2$ μm/s and $K = 270$ μM.[13] (b) The distribution of flow speed at time $t = 6$ hours in the active fluid system for different power-law exponents. Each system has an initial ATP concentration of $C_0 = 8$ mM. The curves are colored based on the n color bar in Panel a. (c) The corresponding squared interface displacement, Δx^2, increases linearly with time, t, for each explored power-law exponent n. The curves are the fitting of $\log(\Delta x^2) = \log(2P_I) + \gamma \log t$ with the interface progression coefficient, P_I, and interface progression exponent, γ, as fitting parameters. Inset: The corresponding interface displacement as a function of time for different power-law exponents. The dots and curves are colored based on the n color bar in Panel a. (d) The corresponding interface progression coefficient, P_I, decreases with power-law exponent n. The red dashed line represents the experimentally measured $P_I = 451 \pm 8$ μm^2/s for 8 mM caged ATP concentration (Fig. 3d). The error bars represent the fitting error in Panel c. Inset: The interface progression exponents remain 1 across explored power-law exponents $n = 0.5$–10. The error bars represent the fitting error in Panel c.

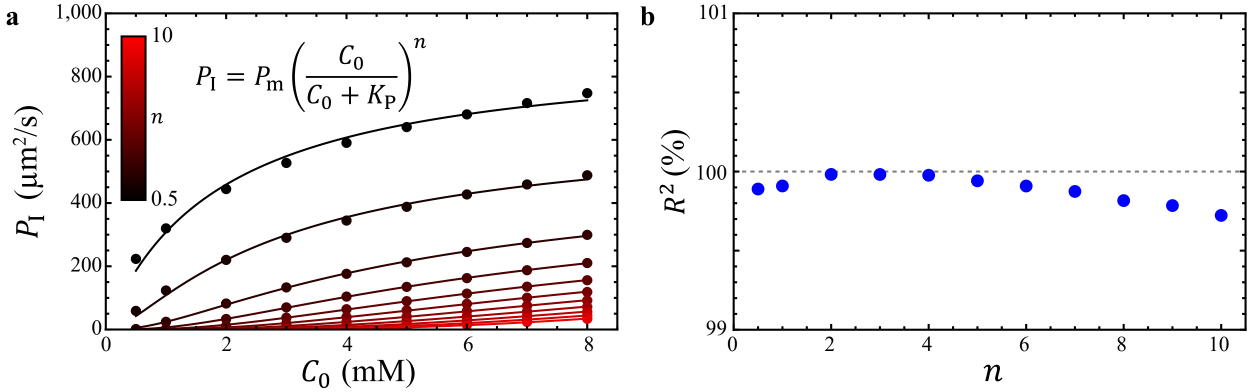

Supplementary Figure 4: (Modeling results) The ATP dependence of interface progression coefficient $P_\mathrm{I}(C_0)$ is inherent from the ATP dependence of the corresponding flow speed of active fluid $\bar{v}(C)$. (a) Interface progression coefficient, P_I, as a function of initial ATP concentration, C_0, for different power-law exponents n. The curves are the fitting to $P_\mathrm{I} = P_\mathrm{m}\{[\mathrm{ATP}]/([\mathrm{ATP}] + K_\mathrm{P})\}^n$ with P_m and K_P as fitting parameters. (b) The goodness of fit, R^2, for power-law exponent data in Panel a.

Supplementary Note 3: Analytical expressions for active-inactive interface progression

Our simple Fick's law-based model shows that the diffusion-like progression of the active-inactive interface is the consequence of diffusive dispersion of ATP (Fig. 3c). This result is based on numerical solutions of the Fick's law equation (Eq. 1); to gain deeper insight into the algebra underlying this modeling result, we consider the dynamics far from the boundary (i.e., discarding Eq. 2). The analytical solution to Fick's law (Eq. 1) subject to an initial step distribution is

$$C(x,t) = \frac{C_0}{2}\,\mathrm{erfc}\left(\frac{x - x_0}{2\sqrt{D\,t}}\right), \qquad 5$$

where x_0 is the initial location of the step. We find the rate of progression of the interface by plugging Supplementary Equation 5 into Eq. 4 and solving for the active-inactive interface displacement Δx. Then the squared interface displacement can be written as:

$$\Delta x^2 = 4\,D\,t\,\left[\mathrm{erfc}^{-1}\left(\frac{2K}{C_0 + 2K}\right)\right]^2 = 2P_\mathrm{I}\,t, \qquad 6$$

where erfc^{-1} is the inverse of the complementary error function and the interface progression coefficient, P_I, is defined as

$$P_\mathrm{I} \equiv 2D\,\left[\mathrm{erfc}^{-1}\left(\frac{2K}{C_0 + 2K}\right)\right]^2. \qquad 7$$

Here, we find that, despite the nonlinearity of the flow speed-ATP conversion (Eq. 4), the interface progression coefficient depends linearly on the diffusion coefficient ($P_\mathrm{I} \propto D$; Supplementary Equation 7) and the progression of the interface remains diffusion-like ($\Delta x^2 \propto t$; Supplementary Equation 6), which is consistent with our numerical results ($\gamma = 1$; Fig. 3c). Moreover, the derived interface progression coefficient $P_\mathrm{I}(C_0)$ is almost identical to the numerical results (magenta curve and red dots in Fig. 3d). We thus reproduce the modeling results (γ and P_I) algebraically.

Supplementary Note 4: Effect of sample container height on the correlation lengths and times of flow velocities

To characterize whether the mixing of our active fluid systems is driven by diffusion-dominated or convection-dominated active transport, we adopt a dimensionless quantity, the Péclet number: $\mathrm{Pe} \equiv \bar{v} l_{\mathrm{c}} / D$, where D is the diffusion coefficient of ATP (Supplementary Note 1), $\bar{v}$ is the flow mean speed of active fluid, and l_{c} is the correlation length of flow velocity of active fluid.[2,3] Determining Pe requires l_{c}, and our previous work shows that increasing the sample container height can increase l_{c}.[14] Thus, we characterize how the correlation lengths and correlation times of active fluid flows depend on the sample container height.

To extract the correlation lengths in our sample, we prepare an active fluid sample with uniform activity, dope the sample with fluorescent tracer particles, and monitor and track these tracers to reveal the flow velocity fields of active fluid in the sample $\mathbf{V}(\mathbf{r}, t)$ as a function of time t (Supplementary Figure 5a). Then we calculate the velocity autocorrelation function,

$$\Psi(\Delta\mathbf{r}, \Delta t) \equiv \int d\mathbf{r}\, dt\, \mathbf{V}(\mathbf{r} + \Delta\mathbf{r}, t + \Delta t) \cdot \mathbf{V}(\mathbf{r} + \Delta\mathbf{r}, t + \Delta t), \qquad 8$$

by deploying the convolution theorem

$$\Psi = \mathcal{F}^{-1}\{\mathcal{F}\{\mathbf{V}\} \cdot \mathcal{F}\{\mathbf{V}\}^{*}\}, \qquad 9$$

where $\mathcal{F}\{\ \}$ represents the Fourier transform, $\mathcal{F}^{-1}\{\ \}$ represents the inverse Fourier transform, and Θ^{*} represents the complex conjugate of any variable Θ. Then we normalize the correlation function as

$$\bar{\Psi}(\Delta\mathbf{r}, \Delta t) = \frac{\Psi(\Delta\mathbf{r}, \Delta t)}{\Psi(\mathbf{0}, 0)}, \qquad 10$$

which allows us to determine the normalized same-time velocity autocorrelation function as $\bar{\Psi}(\Delta\mathbf{r}, 0)$ (Supplementary Figure 5b). To analyze the correlation lengths, we average the correlation function over orientations

$$\bar{\Psi}(\Delta r, 0) = \langle \bar{\Psi}(\Delta\mathbf{r}, 0) \rangle_{|\Delta\mathbf{r}| = \Delta r}, \qquad 11$$

where $\langle\ \rangle_{|\Delta\mathbf{r}| = \Delta r}$ indicates averaging over the same magnitude of spatial displacement $\Delta\mathbf{r}$ (Supplementary Figure 5c). Then we define the correlation lengths l_{c} as the separation distance where the normalized correlation function decays to 0.5: $\bar{\Psi}(l_{\mathrm{c}}, 0) \equiv 0.5$. Repeating the analysis of l_{c} over the samples with heights varying from 60 to 700 μm reveals that the correlation lengths increase from 60 to 210 μm (Supplementary Figure 5d). Given that the correlation lengths also represent the size of vortices in active fluid flow,[15] our analysis suggests that across our explored sample heights the vortices expand by a factor of 3.5.

In addition to correlation lengths, our analysis also allows us to extract the correlation time, τ_{c}, which reveals how rapidly the flow patterns change (i.e., the lifetime of vortices). To determine the correlation time, τ_{c}, we follow a similar analysis except we analyze the normalized same-position temporal autocorrelation function $\bar{\Psi}(\mathbf{0}, \Delta t)$ (Supplementary Figure 5c inset) and define the correlation time τ_{c} as the time lapse when the normalized same-position temporal correlation function decays to 0.5: $\bar{\Psi}(\mathbf{0}, \tau_{\mathrm{c}}) \equiv 0.5$. Our analysis reveals that the correlation time remains ~20 seconds across our explored sample heights (Supplementary Figure 5d inset), which suggests that the sample geometry does not play a significant role in the lifetime of the vortices. Overall, this work shows that increasing the sample container height enlarges

the vortices but does not significantly affect their formation and deformation rates, which aligns with our previous studies about length scales of confined active fluid.[14]

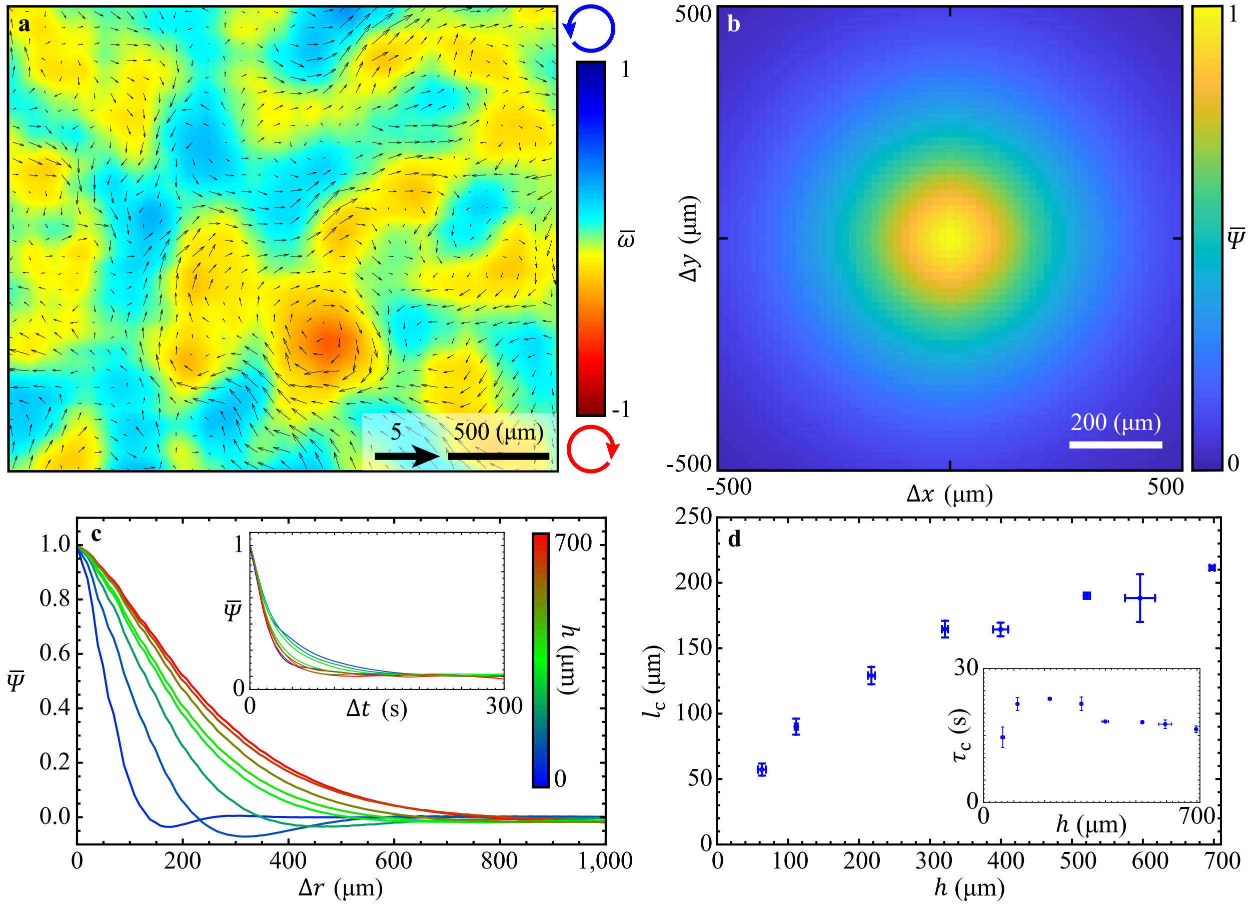

Supplementary Figure 5: (Experimental results) Increasing sample container height increases the correlation lengths of flow velocities but does not significantly influence the correlation times. (a) Normalized velocity field and vorticity color map of active fluid flows in a 700-μm-thick flow cell. The velocity field **V** is normalized by the mean speed of active fluid flow; the vorticity $\omega \equiv [\nabla \times \mathbf{V}]_z$ is normalized by the triple of standard deviation of vorticity, $\overline{\omega} \equiv \omega/[3\ \mathrm{std}(\omega)]$.[16] (b) Map of normalized same-time spatial autocorrelation of flow velocity in the same sample as Panel a. (c) Normalized same-time spatial autocorrelation functions of flow velocity as a function of separation distance (Supplementary Equation 11) for various sample heights. Inset: Normalized same-position temporal autocorrelation functions of flow velocity for various sample heights. (d) The correlation lengths of flow velocity increase with sample height. The error bars represent the standard deviations of two trials. Inset: The correlation times of flow velocity remain nearly invariant (~20 s) for sample heights from 60 to 700 μm.

Supplementary Note 5: Mixing kinematics of activity-uniform active fluid

This work focuses on mixing in active fluid systems with nonuniform distribution of activity. We show that as the Péclet number of a system increases, the progression of the active-inactive interface changes from diffusion-like to superdiffusion-like (Fig. 4b) and the mixing times of suspended fluorescent dyes decrease (Fig. 5c). We decide that it would be elucidating to compare these results with those from active fluid systems with uniform activity distribution. A uniform active fluid system does not have an active-inactive interface, and thus we can not measure γ, but we measure mixing time of suspended fluorescein. We repeat the fluorescein-mixing experiments where the flow speed of active fluid are increased by increasing sample height (Fig. 5), but we perform the experiments in a system with uncaged ATP and thus flow speeds are uniformly distributed throughout the sample (Supplementary Figure 6b inset) and then we analyze the mixing time as a function of mean flow speed and Péclet number (Supplementary Figure 6). Our analysis reveals that the mixing time decreases with increasing Péclet number without a discernible transition, which we expect because a more convective active transport can mix the dye faster. Also, this result is similar to the one in active-inactive fluid systems (Fig. 5c), which suggests that the Péclet number is the controlling parameter for mixing time of suspended components in active fluid systems, regardless of the distribution of activity.

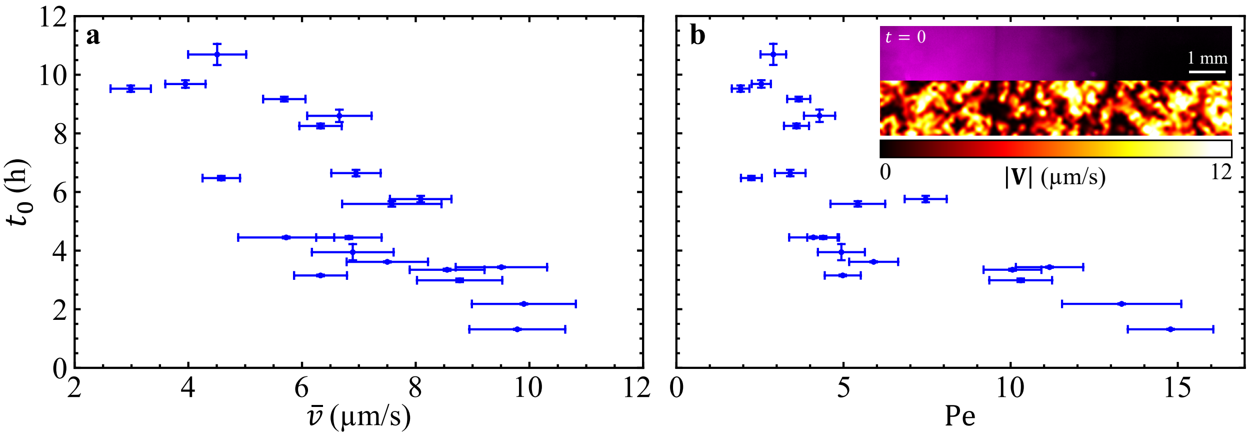

Supplementary Figure 6: (Experimental results) Mixing time of UV-activated fluorescent dyes decreases with increasing Péclet number for activity-uniform active fluid systems. (a) Mixing time as a function of mean speed of active fluid with uniform activity distribution. Accelerating active fluid flows accelerates the mixing process of suspended fluorescein, resulting in shorter mixing time t_0. Each dot represents one experimental measurement. Error bars in t_0 represent the slope fitting error as in Fig. 5b inset, and error bars in $\bar{v}$ represent the standard deviation of time-averaged flow speeds. (b) Mixing time in activity-uniform active fluid systems decreases with increasing Péclet number. The error bars are as in Fig. 5c. Inset: Micrograph of fluorescein uncaged by ultraviolet light exposure (magenta in upper half) and speed map of active fluid (lower half) in the beginning ($t = 0$) of the dye-mixing sample that had mean speed $\bar{v} \approx 9.8 \pm 0.8$ μm/s and mixing time $t_0 = 1.31 \pm 0.02$ h. Note that active fluid has uniform activity (flow speed distribution) in the beginning whereas the uncaged fluorescein is only distributed on one side (left) of the sample.

Supplementary Note 6: Network melting mechanism may slow progression of active-inactive interface

Our active-inactive fluid experiments show that for the active-inactive interface to progress, not only does ATP need to be transported to the inactive fluid region, but also the inactive microtubule network needs to be activated from its inactive state (Supplementary Movie 1). However, inactive microtubule networks behave like an elastic gel because the unfueled kinesin motor dimers are immobile and act as crosslinkers in the microtubule network,[8] and after ATP is transported to the active-inactive interface, it takes time for the fueled motors to fluidize or melt the inactive microtubule network[1] so the interface can progress. Thus, the active-inactive interface is expected to progress more slowly than it would if the network could melt instantly.

To examine this expectation, we analyze the normalized speed profile expected on the basis of ATP distribution and compare them with the measured normalized speed profile. We assume that the ATP is transported by the active fluid in the same way as fluorescein. Also, because ATP and fluorescein are both activated by UV light exposure, we assume that the distribution of dyes is similar to that of activated ATP, which implies that the fluorescein and activated ATP has similar concentration profiles: $C_{\mathrm{ATP}}(x,t)/C_{\mathrm{ATP0}} \approx C_{\mathrm{fluorescein}}(x,t)/C_{\mathrm{fluorescein0}}$ where $C_{\mathrm{X}}(x,t)$ represents the concentration of X and C_{X0} represents the initial concentration of X. We also assume that the gray values in the fluorescein micrographs are proportional to uncaged fluorescein concentration, which implies that the normalized concentration profile of uncaged fluorescein is similar to the normalized profile of gray values (G.V.) in the fluorescein micrographs: $C_{\mathrm{fluorescein}}(x,t)/C_{\mathrm{fluorescein0}} \approx \overline{\mathrm{G.V.}}(x,t)$. Thus, we can deduce the profile of ATP from normalized profile of gray values in fluorescein micrographs: $C_{\mathrm{ATP}}(x,t) \approx C_{\mathrm{ATP0}}\,\overline{\mathrm{G.V.}}(x,t)$. To extract the normalized profile of gray values in fluorescein micrographs, we consider the micrographs of fluorescein near the active-inactive interface (Supplementary Figure 7a). Then we average the gray values of a micrograph vertically to get a profile of gray values, $\mathrm{G.V.}(x,t)$ (Supplementary Figure 7c). To normalize the gray value profile, we adopt the baseline model, which introduces two baselines as the upper and lower references of the gray values: $L_{\mathrm{U}}(x,t)$ and $L_{\mathrm{L}}(x,t)$, where L_{U} is determined by fitting the gray value profile in the active bulk to a line (Supplementary Fig. 6c, magenta curve on top left) and L_{L} is determined by fitting the gray value profile in the inactive bulk to a line (Supplementary Figure 7c, magenta curve on bottom right). Then, the gray value profile can be normalized as

$$\overline{\mathrm{G.V.}}(x,t) \equiv \frac{\mathrm{G.V.}(x,t) - L_{\mathrm{U}}(x,t)}{L_{\mathrm{U}}(x,t) - L_{\mathrm{L}}(x,t)}. \qquad 12$$

This normalization model has been commonly used in the analysis of thermal melting curves of DNA.[17] Here, we adopt this normalization model to reduce the influences of background light and nonuniform illumination in our profile analysis. Once the normalized gray value profile is determined, we can deduce the profile of ATP concentrations (Supplementary Figure 7d). Then we convert the ATP concentration profile to flow speed profile of active fluid with the Michaelis-Menten equation (Eq. 4) followed by normalizing the flow speed profile (blue curve in Supplementary Figure 7e). This is the profile deduced from distribution of uncaged fluorescein. To compare the deduced speed profile with the directly measured speed profile, we analyze the tracer motion at the same time to extract the speed distribution of active fluid flow (Supplementary Figure 7b), average the speed distribution vertically to extract the speed profile, and normalize the profile (red curve in Supplementary Figure 7e). Our analysis shows that the normalized speed profile extracted from tracer motion (red curve in Supplementary Figure 7e) falls behind the profile deduced from uncaged fluorescein distribution (blue curve), which demonstrates that the active-inactive interface progresses more slowly than expected from ATP distribution, and that the network melting plays a role in the progression of the active-inactive interface and can slow down the progression.

The network melting mechanism is absent in our active-fluid hydrodynamic model; the model assumes that the network can melt almost instantly upon arrival of ATP (with negligible warm-up time from the initial development of activity-driven instability in the extensile **Q** field[1,18]), so we expect that the profile discrepancies observed in the experiment (Supplementary Figure 7e) will not exist in our model. To examine the validity of our expectation, we analyze the profiles both directly from flow speed distribution and as calculated on the basis of ATP distribution. In the simulation, we can directly access the ATP distribution (Supplementary Figure 8a), which allows us to determine the corresponding ATP concentration profile (Supplementary Figure 8c inset). Then we convert the concentration profile to speed profile by the Michaelis-Menten equation (Eq. 4) followed by normalization to extract the normalized speed profile (blue curve in Supplementary Figure 8c). To compare this ATP-based profile with the profile from flow speed distribution, we consider the speed map at the same time (Supplementary Figure 8b), average the speed distribution vertically to get the speed profile, and normalize the profile to get the normalized speed profile (red curve in Supplementary Figure 8c). The modeling results show that the ATP-based speed profile and the flow speed-based speed profile nearly overlap across the active-inactive interface, which means that, in the simulation, ATP and activity progress at the same pace. This is not consistent with experimental observations that the progression of activity falls behind ATP (Supplementary Figure 7e). This mismatch between experiments and model results support the existence of a network melting mechanism—in which the network needs to undergo a melting process before it can become fluidized—which is absent in the model.

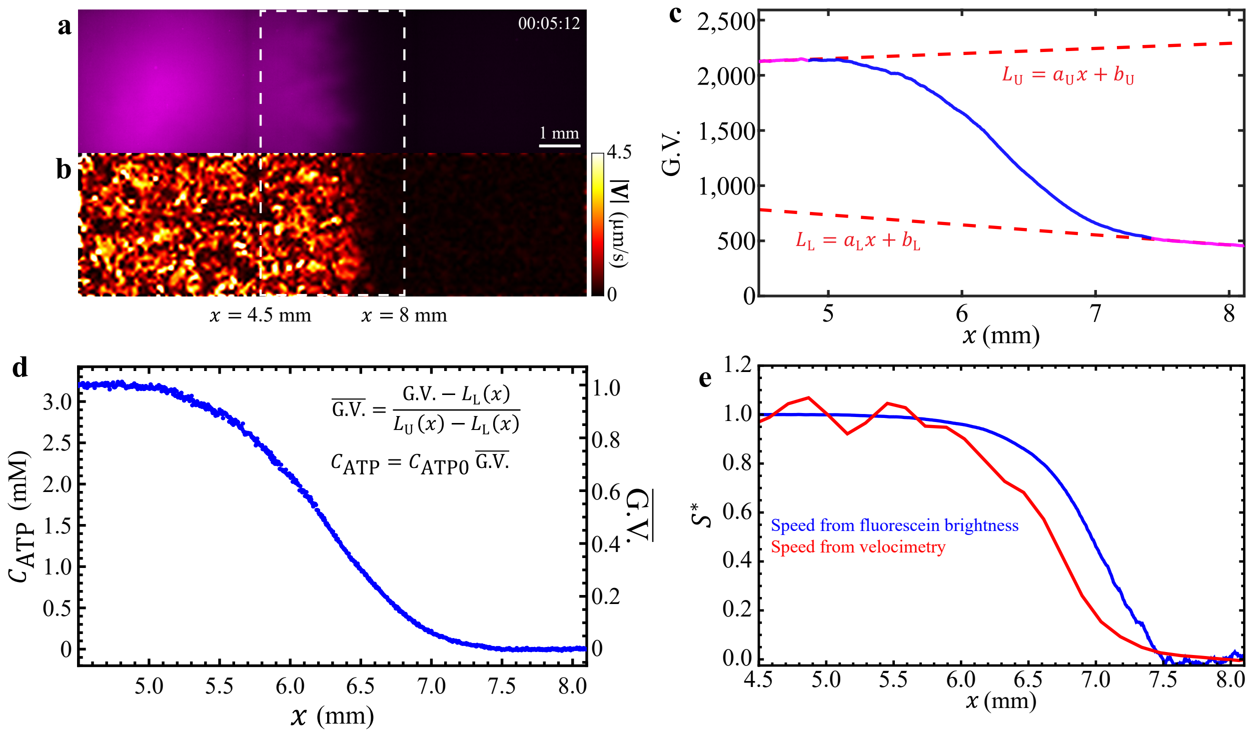

Supplementary Figure 7: (Experimental results) The active-inactive interface progresses more slowly than expected from distribution of ATP. (a) Micrograph of fluorescein (magenta) in the active-inactive sample with an initial ATP concentration of $C_{\text{ATP0}} = 3.2$ mM. The fluorescein is initially caged and thus does not fluoresce; exposure of the left side of the sample to ultraviolet light both activate the microtubule-kinesin fluid and uncaged the fluorescein, allowing it to fluoresce. The white dashed rectangle is the region of interest in the analysis in Panels c-e. The time stamp represents hour:minute:second. (b) The corresponding map of flow speed of active fluid deduced from tracking the motion of tracers. (c) Profile of gray values of the fluorescein micrograph in Panel a. The profile is normalized with a baseline model[17] that includes an upper baseline determined by a line fitting to the gray value profile in the active bulk (the top-left magenta portion of the profile), $L_{\text{U}} = a_{\text{U}} + b_{\text{U}}$ where $a_{\text{U}} = 46$ mm^{-1} and $b_{\text{U}} = 1{,}920$, and a lower baseline determined by a line fitting to the gray value profile in the inactive bulk (the bottom-right magenta portion of the profile), $L_{\text{L}} = a_{\text{L}} + b_{\text{L}}$ where $a_{\text{L}} = -91$ mm^{-1} and $b_{\text{L}} = 1{,}190$. These two baselines serve as the upper and lower references for profile normalization. (d) The gray values are normalized by the baseline model (right axis). The profile of ATP concentrations is deduced by scaling the normalized profile of gray values by the initial concentrations of ATP, C_{ATP0} (left axis). (e) The profile of ATP concentrations is converted to the profile of flow speed by the Michaelis-Menten equation (Eq. 4). The speed profile is normalized (blue curve) as in Fig. 1e and the normalized profile is then compared with the profile measured from the velocimetry of tracers (red curve). The profile analyzed from the velocimetry falls behind the profile deduced from fluorescein brightness, which suggests that the melting mechanism of the crosslinked microtubule network slows the progression of the active-inactive interface.

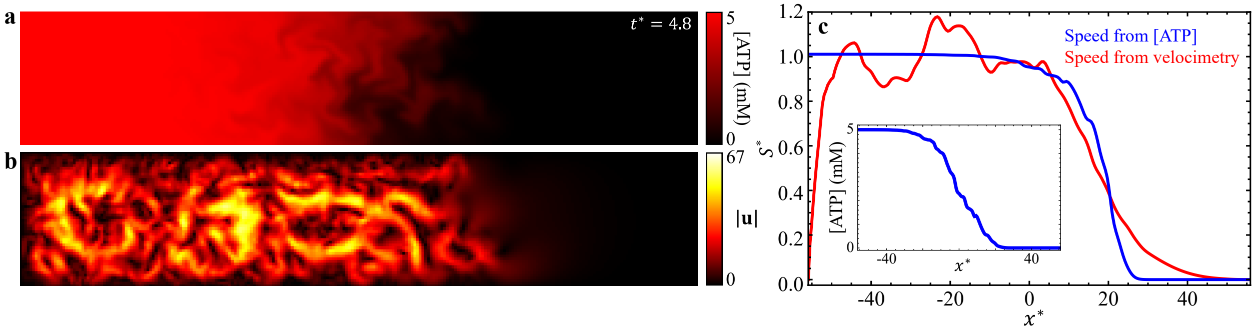

Supplementary Figure 8: (Modeling results) Simulated profiles of active fluid flow and profiles deduced from simulated ATP concentration distribution overlap. (a) Distribution of ATP concentrations in the active fluid simulation (Fig. 6) with $\alpha_0^* = 25$ and $D^* = 2$ at $t^* = 4.8$. (b) The corresponding distribution of flow speed. Note that the flow speed quickly drops to zero (black) as it approaches the boundaries because of the no-slip boundary condition. (c) The corresponding normalized speed profile analyzed from the distribution of flow speed (red curve) and the normalized speed profile deduced from the distribution of ATP (blue curve) nearly overlap at the active-inactive interface. This suggests that, in the simulation, the active fluid is activated almost immediately after the ATP is transported to its location. Inset: The profile of ATP concentrations in Panel a, averaged vertically.

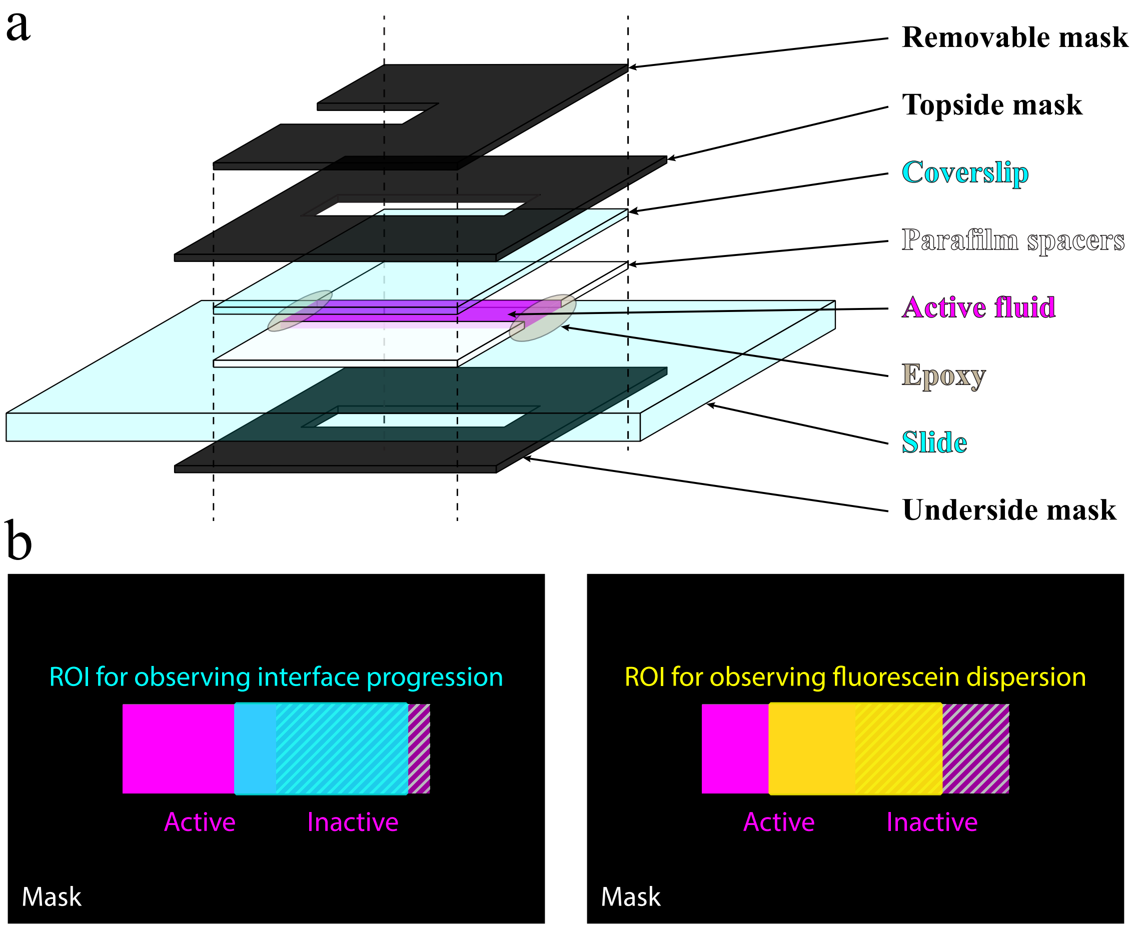

Supplementary Figure 9: Removable masks are used to activate one side of the sample. (a) Active fluid is loaded into a glass flow cell consisting of a polyacrylamide-coated glass slide and coverslip with Parafilm as a spacer and sealed with epoxy. To prepare the active-inactive fluid system, we block one side of the sample (right half) with a removable mask. To prevent UV light from being scattered to the masked region by the epoxy and Parafilm, which can cause unwanted fluid activation in the masked region, we further block the rest of the sample, including epoxy and Parafilm, with 2 masks (one topside and one underside). After the UV exposure, we remove the masks to image the sample with fluorescent microscopy. (b) The sample is 20 mm long, which is wider than the field of view in our microscope even using a 4× objective, so we image 3 to 4 adjacent frames along the flow cell and stitch these frames into one large image. For the experiments monitoring active-inactive fluid interface progression (left), we select the region of interest (ROI) as one quarter of active area and most of the inactive area (cyan rectangle) to observe the progression of the interface (Figs. 1–4). For the experiments monitoring the dispersion of fluorescein (right), we select the ROI as half of the active and half of the inactive regions (yellow rectangle) to observe how one-sided dyes disperse to the rest of the sample (Fig. 5).

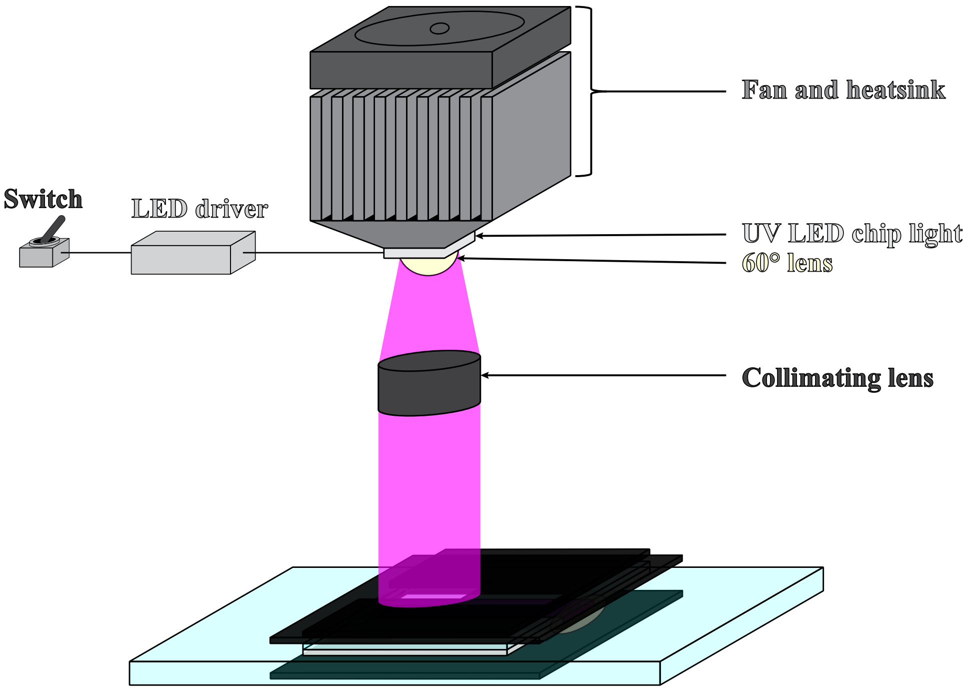

Assembled slide with removable mask

Supplementary Figure 10: Setup to apply UV light to the masked sample.[19] UV light is emitted with a UV LED chip (Amazon, B01DBZK2LM) powered by an LED driver (McMaster, 4305N124) and cooled with a fan-powered heatsink (Amazon, B01D1LD68C). To ensure that the light exposure was consistent across the sample, we parallelize the emitted UV light beams with a 60° lens (part of the heatsink) and a collimating lens (part of the microscope, Nikon, MEA54000).

Supplementary Movie 1: (Experimental results) Mixing of activated and inactive fluids. The fluid contains caged ATP, which can not fuel the kinesin motors until it is uncaged by exposure to ultraviolet light. After one side of the sample is exposed to ultraviolet light, the ATP molecules on that side of the sample are released and can fuel the kinesin motors to drive microtubules and create flows. The activated fluid blends with the inactive fluid until two fluids become one activity-uniform fluid. Cyan fibers are microtubules and red dots are tracers. The time stamp indicates hour:minute:second.

Supplementary Movie 2: (Modeling results) Results of a one-dimensional Fick's law-based model that simulates the mixing of active and inactive fluid under low Péclet number conditions (Pe $\lesssim$ 3). The model describes how ATP distribution evolves from one side of a container to being uniformly distributed (top). The ATP is confined in a segment from $x = 0$ to $x = 20$ mm. The ATP distribution is converted to distribution of active fluid mean speed via Michaelis-Menten kinetics (Fig. 3b). The simulation shows that initially only one side of the system is activated, and then the system evolves toward an activity-uniform state (bottom). Active fluid with a higher initial concentration of ATP (8 mM; red curve) evolves toward an activity-uniform state faster than active fluid with a lower initial concentration of ATP (1 mM; black curve). The time stamp indicates hour:minute:second.

Supplementary Movie 3: (Experimental results) Dispersion of UV-activated fluorescent dyes suspended in inactive (top) and active (bottom) microtubule-kinesin fluid. In the inactive system, the dyes are dispersed only by molecular diffusion, whereas in the active fluid system, the dyes are further transported by active fluid flows and thus disperse through the sample more quickly. Time stamp indicates hour:minute:second.

Supplementary Movie 4: (Modeling results) Simulated maps of ATP concentrations and flow speeds of active fluid for various pairs of dimensionless activity level $\boldsymbol{\alpha_0^*}$ and dimensionless molecular diffusion coefficient $\boldsymbol{D^*}$. In the no-activity system ($\alpha_0^* = 0$; top), dispersion of ATP is driven only by molecular diffusion ($D^* = 16$). When the fluid is activated ($\alpha_0^* = 25$; middle), the chaotic turbulence-like mixing flows are developed to actively transport ATP, which speeds up the ATP dispersion. When the ATP diffusivity is increased ($D^* = 64$, bottom), dispersion of ATP is further accelerated. The simulation captures the roles of ATP diffusion and active fluid-induced convection in dispersing ATP.

Supplementary Movie 5: (Experimental results) Mixing of checkerboard-patterned fluorescein and activity. The checkerboard-pattern distribution of fluorescein and activity is achieved by applying UV light (00:00:12–00:01:06) in a checkerboard pattern to inactive fluid with caged ATP and caged fluorescein. The uncaged fluorescein (magenta in the left panel) is actively transported by flows driven by active microtubule network (cyan fibers in the middle panel) with the same checkerboard pattern of activity and reaches a homogeneously-distributed state in 10 minutes (00:10:30). The right panel represents the merged images of fluorescein (left) and microtubules (middle). The grid size of the checkerboard is $a = 1$ mm. The time stamp indicates hour:minute:second.

Supplementary Movie 6: (Modeling results) Simulation of active and inactive fluid systems with initial checkerboard-patterned ATP. Simulated maps of ATP concentrations (top row) and flow speeds of fluids (bottom row) for active (right column) and inactive (left column) fluid systems where ATP is initially distributed in a checkerboard pattern with a dimensionless grid size of $a^* = 22$. The active fluid system ($\alpha_0^* = 25$; left) actively transports and homogenizes ATP within the dimensionless time $t^* = 10$, while the ATP in the inactive fluid system ($\alpha_0^* = 0$; right), which relies on molecular diffusion ($D^* = 1$) to disperse ATP, does not reach the homogeneous state until the dimensionless time $t^* = 80$.

Supplementary References

1 Najma, B. et al. Dual antagonistic role of motor proteins in fluidizing active networks. *arXiv:2112.11364 [cond-mat.soft]*. (2021).

2 Tuval, I. et al. Bacterial swimming and oxygen transport near contact lines. *Proc Natl Acad Sci U S A* **102**, 2277-2282 (2005).

3 Senoussi, A., Galas, J.-C. & Estevez-Torres, A. Programmed mechano-chemical coupling in reaction-diffusion active matter. *Sci Adv* **7**, eabi9865 (2021).

4 Radomsky, M. L., Whaley, K. J., Cone, R. A. & Saltzman, W. M. Macromolecules released from polymers: Diffusion into unstirred fluids. *Biomaterials* **11**, 619-624 (1990).

5 Saltzman, W. M., Radomsky, M. L., Whaley, K. J. & Cone, R. A. Antibody diffusion in human cervical mucus. *Biophys J* **66**, 508-515 (1994).

6 Soeller, C. et al. Application of two-photon flash photolysis to reveal intercellular communication and intracellular Ca2+ movements. *J Biomed Opt* **8**, 418-427 (2003).

7 Casalini, T., Salvalaglio, M., Perale, G., Masi, M. & Cavallotti, C. Diffusion and aggregation of sodium fluorescein in aqueous solutions. *J Phys Chem B* **115**, 12896-12904 (2011).

8 Gagnon, D. A. et al. Shear-induced gelation of self-yielding active networks. *Phys Rev Lett* **125**, 178003 (2020).

9 Bowen, W. J. & Martin, H. L. The diffusion of adenosine triphosphate through aqueous solutions. *Arch Biochem Biophys* **107**, 30-36 (1964).

10 Schnitzer, M. J. & Block, S. M. Kinesin hydrolyses one ATP per 8-nm step. *Nature* **388**, 386-390 (1997).

11 Coy, D. L., Wagenbach, M. & Howard, J. Kinesin takes one 8-nm step for each ATP that it hydrolyzes. *J Biol Chem* **274**, 3667-3671 (1999).

12 Howard, J., Hudspeth, A. J. & Vale, R. D. Movement of microtubules by single kinesin molecules. *Nature* **342**, 154 (1989).

13 Bate, T. E., Jarvis, E. J., Varney, M. E. & Wu, K.-T. Collective dynamics of microtubule-based 3D active fluids from single microtubules. *Soft Matter* **15**, 5006-5016 (2019).

14 Fan, Y., Wu, K.-T., Aghvami, S. A., Fraden, S. & Breuer, K. S. Effects of confinement on the dynamics and correlation scales in kinesin-microtubule active fluids. *Phys Rev E* **104**, 034601 (2021).

15 Varghese, M., Baskaran, A., Hagan, M. F. & Baskaran, A. Confinement-induced self-pumping in 3D active fluids. *Phys Rev Lett* **125**, 268003 (2020).

16 Wu, K.-T. et al. Transition from turbulent to coherent flows in confined three-dimensional active fluids. *Science* **355**, eaal1979 (2017).

17 Mergny, J.-L. & Lacroix, L. Analysis of thermal melting curves. *Oligonucleotides* **13**, 515-537 (2003).

18 Saintillan, D. & Shelley, M. J. Instabilities, pattern formation, and mixing in active suspensions. *Phys Fluids* **20**, 123304 (2008).

19 Berezney, J., Goode, B. L., Fraden, S. & Dogic, Z. Extensile to contractile transition in active microtubule-actin composites generates layered asters with programmable lifetimes. *Proc Natl Acad Sci U S A* **119**, e2115895119 (2022).
