# Supplementary material for: Self-mixing in microtubule-kinesin active fluid from nonuniform to uniform distribution of activity
Source: Nat Commun. 2022 Nov 2;13:6573. doi: 10.1038/s41467-022-34396-1 (PMC9630547; doi:10.1038/s41467-022-34396-1)
Supplement: Supplementary file 3 — Description of Additional Supplementary Files [file 41467_2022_34396_MOESM3_ESM.pdf]

Description of Additional Supplementary Files

Supplementary Movie 1: **(Experimental results) Mixing of activated and inactive fluids.** The fluid contains caged ATP, which cannot fuel the kinesin motors until it is uncaged by exposure to ultraviolet light. After one side of the sample is exposed to ultraviolet light, the ATP molecules on that side of the sample are released and can fuel the kinesin motors to drive microtubules and create flows. The activated fluid blends with the inactive fluid until two fluids become one activity-uniform fluid. Cyan fibers are microtubules and red dots are tracers. The time stamp indicates hour:minute:second.

Supplementary Movie 2: (Modeling results) Results of a one-dimensional Fick's law-based model that simulates the mixing of active and inactive fluid under low Péclet number conditions ($\mathbf{Pe} \lesssim 3$). The model describes how ATP distribution evolves from one side of a container to being uniformly distributed (top). The ATP is confined in a segment from x = 0 to x = 20 mm. The ATP distribution is converted to distribution of active fluid mean speed via Michaelis-Menten kinetics (Fig. 3b). The simulation shows that initially only one side of the system is activated, and then the system evolves toward an activity-uniform state (bottom). Active fluid with a higher initial concentration of ATP (8 mM; red curve) evolves toward an activity-uniform state faster than active fluid with a lower initial concentration of ATP (1 mM; black curve). The time stamp indicates hour:minute:second.

Supplementary Movie 3: (Experimental results) Dispersion of UV-activated fluorescent dyes suspended in inactive (top) and active (bottom) microtubule-kinesin fluid. In the inactive system, the dyes are dispersed only by molecular diffusion, whereas in the active fluid system, the dyes are further transported by active fluid flows and thus disperse through the sample more quickly. Time stamp indicates hour:minute:second.

Supplementary Movie 4: (Modeling results) Simulated maps of ATP concentrations and flow speeds of active fluid for various pairs of dimensionless activity level $\boldsymbol{\alpha_0^*}$ and dimensionless molecular diffusion coefficient $\boldsymbol{D^*}$. In the no-activity system (α_0^* = 0; top), dispersion of ATP is driven only by molecular diffusion (D^* = 16). When the fluid is activated (α_0^* = 25; middle), the chaotic turbulence-like mixing flows are developed to actively transport ATP, which speeds up the ATP dispersion. When the ATP diffusivity is increased (D^* = 64, bottom), dispersion of ATP is further accelerated. The simulation captures the roles of ATP diffusion and active fluid-induced convection in dispersing ATP.

Supplementary Movie 5: (Experimental results) Mixing of checkerboard-patterned fluorescein and activity. The checkerboard-pattern distribution of fluorescein and activity is achieved by applying UV light (00:00:12–00:01:06) in a checkerboard pattern to inactive fluid with caged ATP and caged fluorescein. The uncaged fluorescein (magenta in the left panel) is actively transported by flows driven by active microtubule network (cyan fibers in the middle panel) with the same checkerboard pattern of activity and reaches a homogeneously-distributed state in 10 minutes (00:10:30). The right panel represents the merged images of fluorescein (left) and microtubules (middle). The grid size of the checkerboard is a = 1 mm. The time stamp indicates hour:minute:second.

Supplementary Movie 6: (Modeling results) Simulation of active and inactive fluid systems with initial checkerboard-patterned ATP. Simulated maps of ATP concentrations (top row) and flow speeds of fluids (bottom row) for active (right column) and inactive (left column) fluid systems where ATP is initially distributed in a checkerboard pattern with a dimensionless grid size of α^* = 22. The active fluid system (α_0^* = 25; left) actively transports

and homogenizes ATP within the dimensionless time t^* = 10, while the ATP in the inactive fluid system (α_0^* = 0; right), which relies on molecular diffusion (D^* = 1) to disperse ATP, does not reach the homogeneous state until the dimensionless time t^* = 80.
